# Automated Detection of Tailing Impoundments in Multi-Sensor High-Resolution Satellite Images Through Advanced Deep Learning Architectures

**DOI:** 10.3390/s25144387

**Published:** 2025-07-14

**Authors:** Lin Qin, Wenyue Song

**Affiliations:** 1 School of Resources and Environmental Engineering, Hefei University of Technology, Hefei 230009, China; 2021020022@mail.hfut.edu.cn; 2 School of Artificial Intelligence, Anhui University, Hefei 230039, China

**Keywords:** tailing impoundment, object detection, satellite image, deep learning

## Abstract

Accurate spatial mapping of Tailing Impoundments (TIs) is vital for environmental sustainability in mining ecosystems. While remote sensing enables large-scale monitoring, conventional methods relying on single-sensor data and traditional machine learning-based algorithm suffer from reduced accuracy in cluttered environments. This research proposes a deep learning framework leveraging multi-source high-resolution imagery to address these limitations. An upgraded You Only Look Once (YOLO) model is introduced, integrating three key innovations: a multi-scale feature aggregation layer, a lightweight hierarchical fusion mechanism, and a modified loss metric. These components enhance the model’s ability to capture spatial dependencies, optimize inference speed, and ensure stable training dynamics. A comprehensive dataset of TIs across varied terrains was constructed, expanded through affine transformations, spectral perturbations, and adversarial sample synthesis. Evaluations confirm the framework’s superior performance in complex scenarios, achieving higher precision and computational efficiency than state-of-the-art detectors.

## 1. Introduction

Tailing Impoundments (TIs) represent specialized containment structures designed to store mineral processing byproducts and industrial waste materials extracted from both metallic and non-metallic ore deposits. The systematic monitoring of these artificial reservoirs plays a crucial role in environmental protection and geohazard mitigation within mining regions [1]. Conventional monitoring approaches typically rely on labor-intensive field surveys combined with visual image interpretation techniques [2,3]. However, the frequently remote locations of these containment facilities make ground-based investigations particularly challenging in terms of time and resource requirements. Recent developments in satellite imaging technology have enabled the feasibility of automated TI identification through remote sensing analysis.

Traditional machine learning approaches have demonstrated widespread application in TI detection. Previous research by Mezned et al. [4] utilized fused Landsat-7 and SPOT-5 imagery combined with linear spectral unmixing techniques to delineate areas affected by mining residues. Another study by Ma et al. [5] introduced a specialized iron content index that enabled TI identification through entropy-based differentiation between target areas and their surroundings. Hao et al. [6] further advanced this field by developing a multi-index framework for TI recognition using Landsat-8 data. While these methods have shown value, they remain constrained by their dependence on manual feature engineering processes. The inherent variability in TI characteristics (including spectral signatures, geometric configurations, and surrounding landscapes) significantly limits the scalability of these conventional approaches for large-area monitoring applications.

The emergence of deep learning methodologies has revolutionized various computer vision domains [7,8,9,10], offering new possibilities for TI detection. Initial applications of these techniques to TI identification appeared in the work of Lyu et al. [11], who implemented a U-Net architecture for analyzing Chinese GF-6 satellite imagery. Subsequent improvements were achieved by Yan et al. [12] through an optimized R-CNN framework trained on expanded datasets, yielding more reliable detection performance.

Despite these advances, several critical challenges persist in deep learning-based TI detection. The primary difficulty stems from the complex visual environments surrounding TI s, where natural water bodies and other artificial reservoirs often exhibit similar spectral properties to tailing impoundments. Furthermore, the heterogeneous composition of stored materials leads to significant intra-class variation in TI appearance. Current limitations are exacerbated by insufficient training data diversity, with most studies employing limited datasets from single imaging sensors. These factors collectively contribute to elevated false positive rates in complex scenes and poor model generalization across different data sources [13,14]. Additional practical constraints include substantial computational requirements and slow training processes characteristic of deep neural networks.

To address these challenges, this study presents an enhanced YOLOv7-based [15] TI detection architecture which is trained by multi-source satellite images. This work incorporates several key parts: (1) comprehensive dataset collection spanning diverse geographical contexts and imaging conditions, and strategic data augmentation to enhance sample variability; (2) a Multi-Scale Feature Aggregation (MSFA) layer promoting the network’s effectiveness in capturing global information hidden in the images; (3) a Lightweight Hierarchical Fusion (LHF) mechanism to help the model to keep balance between detection precision and inference efficiency; (4) a refined loss function to prevent optimization divergence.

Specifically, the main innovations of this study are as follows:

(1) Current research on deep learning-based TI identification remains relatively scarce, with existing approaches demonstrating limited robustness primarily due to inadequate training data diversity. Our methodology addresses this limitation through comprehensive multi-sensor image collection across varied environments, combined with systematic data augmentation techniques. This enriched training approach enables our enhanced YOLOv7 architecture to achieve superior detection performance with minimal false positives, even when processing imagery containing complex background interference.
(2) The core feature extraction network incorporates our novel Multi-Scale Feature Aggregation (MSFA) layer, designed to simultaneously streamline the model architecture while improving its capacity to integrate comprehensive contextual information about target objects. This dual optimization enhances both computational efficiency and detection performance.
(3) Within the feature fusion network, we implement a Lightweight Hierarchical Fusion (LHF) mechanism that significantly improves the model’s ability to combine multi-scale features efficiently. This lightweight design maintains computational economy while enabling more precise characterization of target attributes through optimized information integration.
(4) To resolve training instability issues inherent in conventional YOLOv7 implementations, we introduce a sophisticated loss function modification. This adaptation not only prevents optimization divergence but also substantially reduces the time required for model convergence during the training phase.

The subsequent sections are organized as follows: Section 2 describes the multi-sensor dataset and sample generation procedures; Section 3 presents the modified YOLOv7 architecture; Section 4 evaluates experimental results; and Section 5 provides concluding remarks.

## 2. Datasets and Samples

### 2.1. Datasets

The experimental framework of this research utilizes multi-sensor high-resolution satellite imagery to develop and evaluate our enhanced YOLOv7-based TI detection system. The primary data sources comprise China’s Gaofen satellite series (including Gaofen 1 through 1D and Gaofen 6 platforms) along with ZY-3 satellite acquisitions, with geographical coverage concentrated in China’s Shaanxi and Gansu provinces. Comprehensive sensor specifications and imaging parameters are detailed in Table 1.

**Figure 1:**
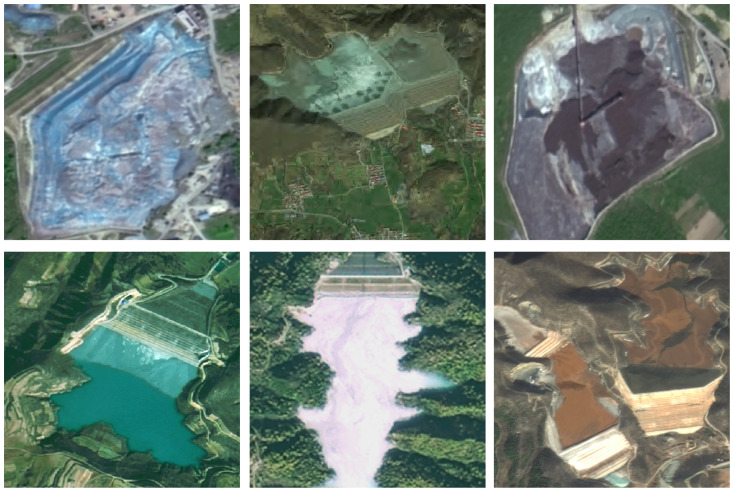
Examples of TI targets on the sample images.

As illustrated in Figure 1, the visual complexity of tailing impoundment environments presents significant analytical challenges. Several factors contribute to this complexity, including the following: substantial variation in surrounding terrain and land cover characteristics; considerable differences in pond dimensions and spatial orientations; diverse morphological configurations based on geographical setting. To ensure robust model performance, our training protocol emphasizes the inclusion of representative samples capturing this natural variability. Specifically, our curated dataset encompasses three fundamental TI classifications: Valley-adjacent TIs (V-TIs), Slope-associated TIs (S-TIs), and Flat terrain TIs (F-TIs).

### 2.2. Sample Generation

The collected multi-source imagery was processed into 2166 standardized image patches, each measuring 1024 × 1024 pixels, containing a total of 2537 identified TI instances, among which, there are 1719 V-TIs, 606 S-TIs, and 21 F-Tis, respectively. This stratified distribution across varied topographic settings ensures comprehensive representation of different tailing impoundment configurations, which is crucial for developing generalizable deep learning models.

Effective deep learning applications require extensive training data, yet tailing impoundments’ geographical sparsity makes large-scale manual annotation impractical. Recognizing that impoundment characteristics vary significantly with acquisition parameters (including sensor type, temporal factors, and viewing geometry), we implemented multiple data augmentation techniques to artificially enhance sample diversity. This approach addresses the fundamental challenge of limited training data availability while accounting for the natural variability in tailing impoundment appearances across different imaging conditions.

(1) Spatial Transformation Augmentation: To account for size and orientation variations, we applied geometric transformations to each sample. These spatial modifications help the model recognize TIs regardless of their dimensional or rotational differences in satellite imagery. The transformation matrix governing this process is mathematically represented as follows:

(1) $$\left[\begin{array}{c} x_{2}\\ y_{2}\\ 1 \end{array}\right]=\left[\begin{array}{ccc} t_{11} & t_{12} & \Delta x\\ t_{21} & t_{22} & \Delta y\\ 1 & 0 & 1 \end{array}\right]\left[\begin{array}{c} x_{1}\\ y_{1}\\ 1 \end{array}\right]$$

where $\left(x_{1},y_{1}\right)$ and $\left(x_{2},y_{2}\right)$ denotes the pixels’ coordinates in images before and after transformation, respectively. Different affine transformations can be applied by using different affine transformation functions, thus obtaining sufficient TI samples with different sizes and different orientations.

(2) Spectral Variation Augmentation: Recognizing that different mineral compositions produce varying spectral signatures, we performed color space manipulations. This involved systematic adjustments to RGB channel values, modifying brightness levels, contrast ratios, and color saturation intensities. Such variations improve the model’s ability to identify TIs under different illumination conditions and mineralogical compositions.

(3) Contextual Adversarial Augmentation: Building upon Ghiasi et al.’s methodology [16], we developed a specialized copy-paste technique to address classification challenges. Our implementation first identifies problematic false positives through preliminary model testing. These challenging negative samples then receive carefully cropped TI regions from verified positive samples, creating synthetic training examples that specifically target classification weaknesses.

Through this comprehensive augmentation framework, we successfully expanded our dataset to 69,312 sample images. Following standard machine learning practice, we partitioned this enhanced dataset into training (70%) and testing (30%) subsets using randomized allocation.

## 3. Method

The YOLO architecture performs object detection through probabilistic anchor box generation, where each box represents a potential target with associated confidence scores. Among contemporary object detection algorithms, YOLOv7 [15] stands out through its sophisticated network design and intelligent model scaling approach. This version demonstrates superior performance in achieving an optimal balance between processing speed and detection accuracy when compared to its predecessors [17,18,19,20,21,22]. Given our specific requirements for handling intricate remote sensing environments while maintaining computational efficiency, YOLOv7 serves as the core architecture for our tailing impoundment detection system.

**Figure 2:**
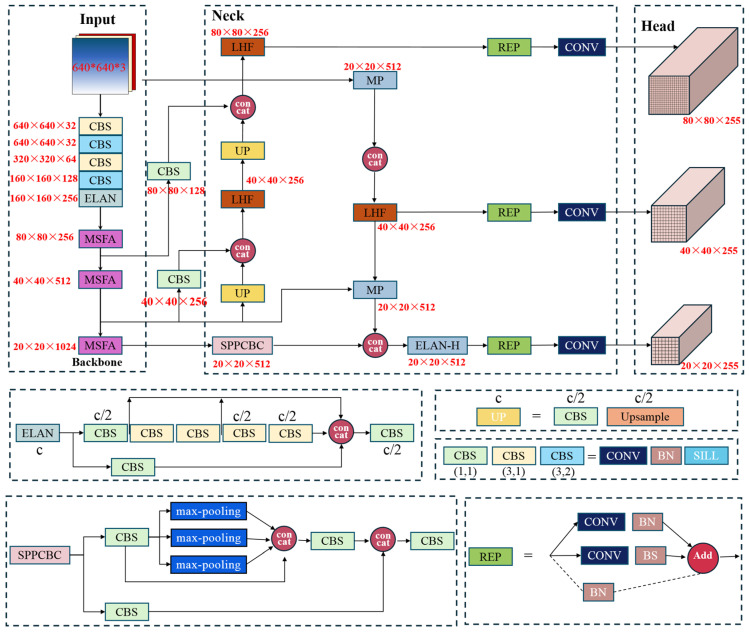
Flowchart of the proposed TI detection model.

Figure 2 illustrates the comprehensive structure of our modified YOLOv7 framework. The system incorporates several key enhancements. The MSFA component is integrated into the backbone network to enhance multi-scale feature extraction. Complementary to this, we employ strategic cross-layer pooling operations to improve information flow between network levels, thereby boosting overall detection capability. The LHF mechanism is implemented in the neck part to address the critical challenges came from the complex environmental contexts in satellite imagery and the substantial appearance variations among TIs. This module optimizes the trade-off between computational demands and detection performance. The system utilizes Distance-IoU (DIoU) metrics for target verification and loss calculation. This evaluation framework enables dynamic model refinement during training by providing more accurate spatial relationship assessments between predicted and actual targets.

**Figure 3:**
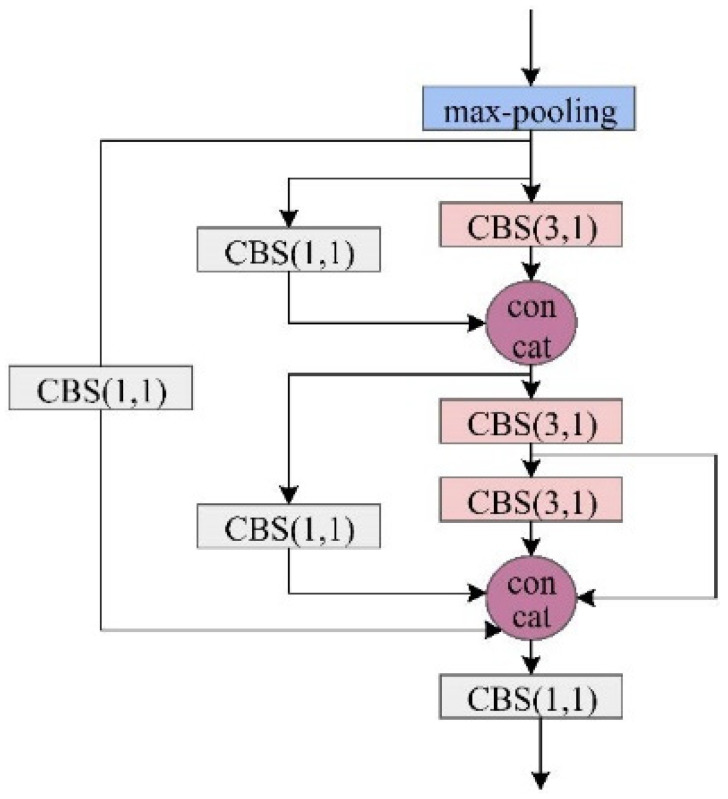
The proposed MSFA architecture.

(1) MSFA Architecture: The conventional approach of feeding raw images directly into the backbone network often results in suboptimal utilization of contextual information, particularly detrimental for target recognition in cluttered environments. To address this limitation, we developed the MSFA module (Figure 3) specifically designed to enhance global feature extraction.

The module employs a multi-stage processing pipeline. Initial downsampling is achieved through max-pooling operations, serving dual purposes of reducing computational complexity while preserving critical spatial information that might otherwise be lost through excessive convolutional layers. The processed features then undergo multi-scale analysis using strategically sized Convolutional Kernels (Conv-Ks). Larger Conv-Ks establish cross-channel feature relationships, and specialized Conv-Ks extract high-level semantic patterns. Then, the combined outputs create an expanded receptive field. The final stage employs 1 × 1 convolutions for dimensional consistency, effectively consolidating the enriched feature representations while maintaining computational efficiency.

**Figure 4:**
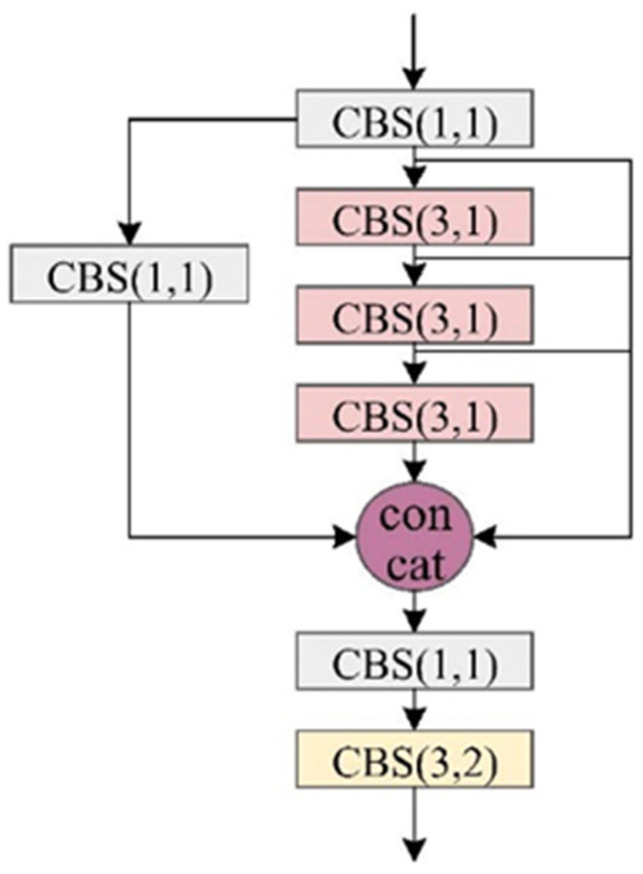
The proposed LHF module.

**Figure 5:**
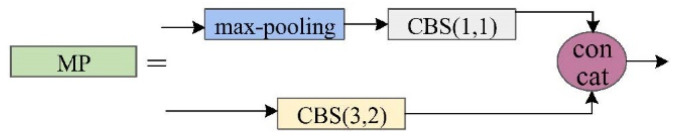
Cross-layer association module.

(2) LHF Unit: The LHF module (Figure 4) represents a crucial optimization of the Neck Feature Fusion Network (NFFN), specifically designed to achieve lightweight model architecture without compromising performance. A specialized cross-level connectivity framework establishes dynamic links between the standard YOLOv7 backbone and its tiny version counterpart [15]. This integration, mediated through our cross-layer association unit (Figure 5), intelligently weights and balances multi-scale feature representations extracted from preceding network layers. The module employs strategic max-pooling operations during feature propagation from backbone to neck networks. This approach is helpful to maintain critical spatial context throughout the network and reduces computational overhead while preventing information degradation. The complete LHF implementation achieves superior computational efficiency while enhancing the model’s ability to integrate and process multi-scale feature information—a critical requirement for accurate target detection in complex remote sensing environments.

(3) Enhanced Loss Function Optimization: A key enhancement in our TI detection framework involves replacing the conventional Intersection over Union (IoU) [23] metric with the Distance-IoU (DIoU) [24] for loss computation during model training. The traditional IoU has some notable drawbacks [25]: first, it is highly sensitive to the scale of objects, making it less reliable for comparing small objects with low overlap; secondly, it only measures overlap and ignores the spatial distance between non-overlapping bounding boxes, which may make two boxes far apart have the same IoU (IoU = 0) as boxes slightly apart, failing to reflect localization accuracy; thirdly, when boxes do not overlap (IoU = 0), the gradient becomes zero, halting training and slowing convergence. DIoU offers superior performance in bounding box regression by incorporating three critical geometric factors. First, it employs centroid distance to measure the spatial separation between predicted and ground-truth box centers. Secondly, it considers the overlap area to maintain the original IoU’s overlap ratio evaluation. Thirdly, it uses aspect ratio to account for scale discrepancies between target and prediction.

DIoU is calculated as

(2) $$\mathrm{DIoU}=\frac{P\cap T}{P\cup T}-\frac{d^{2}(c^{p},c^{t})}{l^{2}}$$

where *P* and *T* denote the areas of the predicted anchor and the true anchor, respectively; *l* is the diagonal distance of the minimum bounding rectangle including the predicted and the true boxes; $c^{p}$ and $c^{t}$ represents, respectively, the center of the output box and the reference box, and ρ is the Euclidean distance between these two boxes.

## 4. Experimental Part

### 4.1. Evaluation Metric

The detection capability of our enhanced YOLOv7 framework for TI identification in multi-sensor remote sensing data was assessed using three statistical measures: the overall accuracy (OA), the F1-score, and the mean Average Precision (mAP) [26].

OA represents the proportion of correctly classified instances relative to the total sample size. Given the True Positive detections (TP), the True Negative exclusions (TN), the False Positive errors (FP) and the False Negative omissions (FN), OA is mathematically expressed as follows:

(3) $$\mathrm{OA}=\frac{\mathrm{TP}+\mathrm{TN}}{\mathrm{TP}+\mathrm{FP}+\mathrm{FN}+\mathrm{TN}}$$

F1-score is a harmonic mean combines precision and recall metrics:

(4) $$F1=\frac{2\cdot \mathrm{Precision}\cdot \mathrm{Recall}}{\mathrm{Precision}+\mathrm{Recall}}$$

where

(5) $$\mathrm{Precision}=\frac{\mathrm{TP}}{\mathrm{TP}+\mathrm{FP}}$$

and

(6) $$\mathrm{Recall}=\frac{\mathrm{TP}}{\mathrm{TP}+\mathrm{FN}}$$

mAP provides a robust measure of a model’s accuracy by considering both precision and recall across different IoU confidence thresholds, which is calculated as

(7) $$\mathrm{mAP}=\frac{1}{N}\sum_{i=1}^{N}{\mathrm{AP}}_{i}$$

where *N* denotes the number of classes, and AP is defined as the Precision-Recall (P-R) curve, which summarizes model performance across different confidence thresholds (such as, mAP50 and mAP75).

For computational efficiency analysis, we employed two additional metrics: the number of parameters (Par) and the Frames Per Second (FPS) [27]. Par is given by

(8) $$\mathrm{Par}=Q^{2}\cdot C_{1}\cdot C_{2}$$

where the total trainable parameter *Q* means the size of Conv-Ks; $C_{1}$ and $C_{2}$ are, respectively, the numbers of input and output channels.

Given the networks’ processing time t1 and the post-processing time t2, FPS is defined as

(9) $$\mathrm{FPS}=\frac{1}{t1+t2}$$

### 4.2. Ablation Experiments

**Figure 6:**
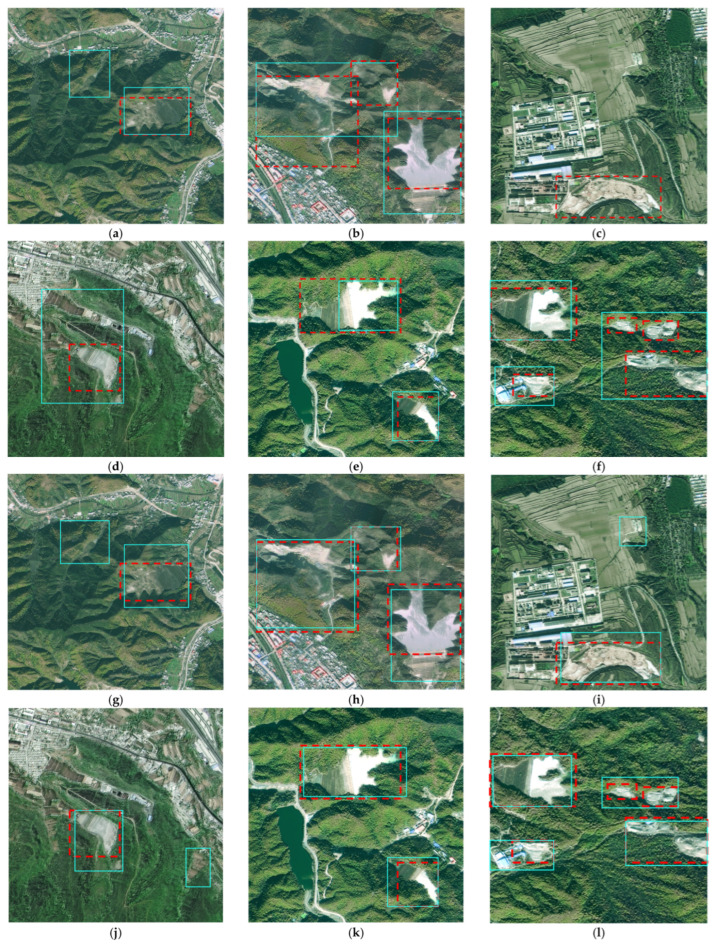

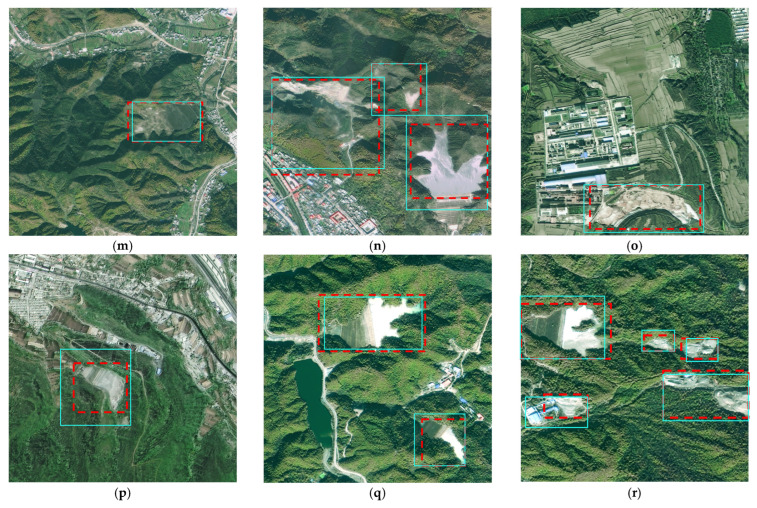
TI detection results by the baseline YOLOv7 model and the proposed model (red boxes: reference anchor boxes; green boxes: predicted anchor boxes). (**a**,**b**) The predictions by the baseline YOLOv7 on the V-TI objects without sample augmentation. (**c**,**d**) The predictions by the baseline YOLOv7 on the S-TI objects without sample augmentation. (**e**,**f**) The predictions by the baseline YOLOv7 on the F-TI objects without sample augmentation. (**g**,**h**) The predictions by the baseline YOLOv7 on the V-TI objects with sample augmentation. (**i**,**j**) The predictions by the baseline YOLOv7 on the S-TI objects with sample augmentation. (**k**,**l**) The predictions by the baseline YOLOv7 on the F-TI objects with sample augmentation. (**m**,**n**) The predictions by the enhanced model on the V-TI objects. (**o**,**p**) The predictions by the enhanced model on the S-TI objects. (**q**,**r**) The predictions by the enhanced model on the F-TI objects.

To systematically evaluate the performance improvements achieved through our architectural innovations (including the MSFA module, LHF component, and optimized loss function), we conducted a series of ablation experiments. Figure 6 presents side-by-side comparisons of detection outputs between our enhanced architecture and the unmodified YOLOv7 baseline model with and without sample augmentation approaches. Table 2 provides comprehensive metrics quantifying the performance differences across multiple evaluation dimensions.

The experimental results demonstrate significant improvements in both detection quality and processing efficiency. The enhanced model exhibits robust performance across all evaluation criteria. It obtains precise target identification (high true positive rate) and accurate bounding box localization (spatial alignment). Moreover, the enhanced model maintains consistent performance even in challenging scenarios with complex terrain environments and presence of spectrally similar non-target features. On the contrary, the baseline YOLOv7 implementation shows notable limitations when processing images with heterogeneous backgrounds and scenes containing deceptive non-target objects. The data in Table 2 further confirms that the proposed model has achieved an increase of 5.2% in OA and an increase of 6.7% in F1-score with regard to the YOLOv7 baseline model with sample augmentation. It can be found that the sample augmentation approaches boost the TI detection precision. In addition, each architectural component in the model has indeed improved the detection results.

### 4.3. Comparison Experiments

To comprehensively evaluate our model’s performance advantages, we conducted rigorous benchmarking against state-of-the-art object detection architectures, including the classical two-stage detector R-CNN [12]; the Version 5, 6 and 8 of YOLO variants [21,22,28]; and the DEtection TRansformer (DETR) [29]. Table 3 and Table 4 present the systematic comparison when setting different IoU thresholds for detection.

The evaluation results demonstrate significant variations in performance characteristics across different detection architectures. R-CNN framework exhibits substantial computational overhead due to its extensive parameterization and delivers suboptimal detection accuracy despite complex architecture. YOLOv5 shows higher annotation precisions than R-CNN but it fails to maintain optimal speed-accuracy balance. YOLOv6 architecture further improves the annotation precisions with lightweight networks and higher inference efficiency. The YOLOv8 architecture represents the current state-of-the-art in the YOLO family, demonstrating significant advancements over previous iterations. Compared with YOLOv8, DETR obtains lower detection precision but shows higher computational efficiency. In addition, we can also notice that, as the IoU threshold is set more strictly, the accuracies of object detection in the different models decrease. But the proposed model still outperforms other models when setting stricter threshold.

The quantitative results validate our architectural innovations in achieving both precision and efficiency goals for practical deployment scenarios. This balanced performance profile makes our solution particularly suitable for large-scale remote sensing applications requiring real-time processing capabilities.

**Figure 7:**
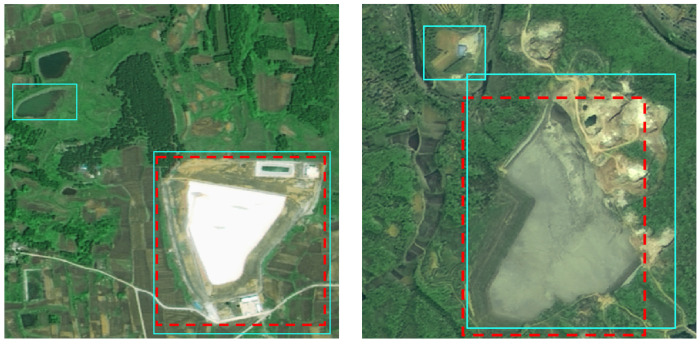
Examples of false predictions on the F-TIs targets provided by the proposed method (red boxes: reference anchor boxes; green boxes: predicted anchor boxes).

Table 5 presents the classification performance of our detection framework across various topographic categories of tailing impoundments, revealing superior accuracy for V-TIs compared to S-TIs and F-TIs. This performance disparity primarily stems from dataset imbalance, with mountainous samples outnumbering other instances, compounded by the prevalence of spectrally similar features in plains regions. Figure 7 illustrates characteristic misclassifications where some ephemeral water bodies and bare lands are incorrectly identified as TIs due to comparable visual characteristics in both spectral reflectance and geometric patterns, highlighting the current limitations in distinguishing subtle feature differences within homogeneous landscapes.

**Figure 8:**
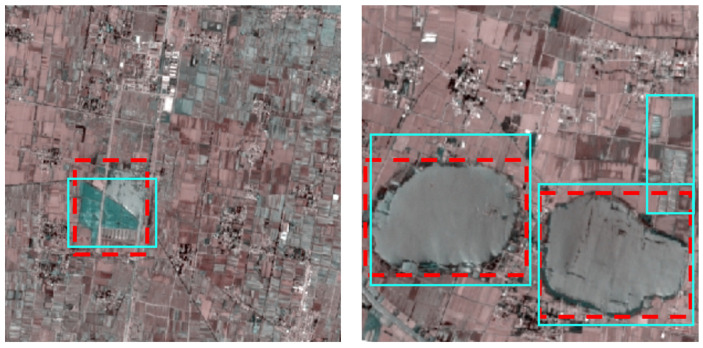
TI detection results of the proposed model on Sentinel-2 images (red boxes: reference anchor boxes; green boxes: predicted anchor boxes).

To inspect the generalization capability of the proposed detection method in detecting TI targets across different sensor and different region, we display the detection results on Sentinel-2 images acquired in Anhui Province, China, in Figure 8 and showcase the assessment values in Table 6. It can be observed, although the assessment values are notable lower than that on Gaofen and ZY-3 satellite images, the detection precision is generally acceptable.

## 5. Conclusions

The automated detection of Tailing Impoundments (TIs) in satellite imagery presents both significant value and notable technical challenges. The complexity arises from several factors: intricate surrounding landscapes that vary substantially across mining regions, and numerous natural features (such as water bodies and bare soil patches) that closely resemble TIs in spectral and textural characteristics. These challenges necessitate the development of highly robust algorithms capable of maintaining accuracy across diverse environmental conditions. Deep learning approaches offer particular promise for this application due to their exceptional pattern recognition capabilities and scalability for large-area monitoring once properly trained. However, current implementations face three primary limitations: (1) reliance on restricted training datasets with homogeneous backgrounds from single sensors, leading to elevated false positive rates in complex scenes and poor cross-sensor generalization; (2) excessive computational requirements during training due to model complexity; and (3) inefficient inference speeds that hinder operational deployment. These constraints highlight the need for optimized architectures that balance accuracy with computational efficiency while accommodating multi-source imagery. Recent advances in lightweight network design and transfer learning techniques may provide pathways to address these challenges, potentially enabling more practical TI monitoring solutions for environmental management applications.

To address these challenges, this study develops an enhanced YOLOv7-based framework for TI detection in multi-sensor satellite imagery. Our approach incorporates a comprehensive training dataset comprising high-resolution images from diverse geographic contexts and sensor systems, augmented through advanced techniques to ensure robust feature learning; then, the architectural improvements including multi-scale feature aggregation and lightweight hierarchical fusion mechanisms are made to optimize the accuracy-efficiency trade-off, and a Distance-IoU loss function is implemented to improve bounding box regression stability. Experimental validation across varied landscapes demonstrates the framework’s superior performance, achieving both precise localization and real-time processing capabilities.

While the current study demonstrates promising results, it is important to acknowledge that our training data primarily consists of imagery from China’s Gaofen satellite constellation. This sensor-specific focus could potentially constrain the model’s ability to generalize across different satellite platforms with varying spectral and spatial characteristics. To address this limitation, our future research will focus on two key improvements: the first one is to expand the training dataset to incorporate multi-sensor, high-resolution imagery from diverse international satellite systems, and the second one is to develop optimized training protocols to enhance both model generalization and computational efficiency.

## Figures and Tables

**Table 1:** Lists of the TI detection datasets.

Parameters	Lists
satellites	Gaofen series and ZY-3
spatial Resolution	better than 3 m
geographical coverage	Shaanxi and Gansu Provinces, China
acquisition time	2017/2019

**Table 2:** Ablation experimental results.

Model	OA	F1-Score	mAP50	mAP75
the baseline YOLOv7 (without sample augmentation)	0.772	0.808	0.451	0.398
the baseline YOLOv7 (with sample augmentation)	0.851	0.830	0.523	0.401
YOLOv7 with MSFA	0.884	0.871	0.588	0.487
YOLOv7 with LHF	0.862	0.887	0.539	0.440
YOLOv7 with DIoU loss	0.866	0.859	0.540	0.416
the enhanced model	0.903	0.897	0.646	0.555

**Table 3:** TI detection performances of the different models when setting the IoU threshold as 0.5.

Model	OA	F1-Score	mAP	Par	EPS
R-CNN	0.764	0.709	0.447	141.3 M	17.5
YOLO Version 5	0.820	0.789	0.469	82.1 M	50.1
YOLO Version 6	0.844	0.823	0.504	69.8 M	72.4
YOLO Version 8	0.891	0.856	0.592	66.6 M	41.3
DETR	0.855	0.840	0.531	41.2 M	48.1
the proposed one	0.903	0.897	0.646	17.3 M	137.7

**Table 4:** TI detection performances of the different models when setting the IoU threshold as 0.75.

Model	OA	F1-Score	mAP	Par	EPS
R-CNN	0.640	0.512	0.300	141.3 M	17.5
YOLO Version 5	0.711	0.645	0.383	82.1 M	50.1
YOLO Version 6	0.777	0.712	0.424	69.8 M	72.4
YOLO Version 8	0.800	0.787	0.498	66.6 M	41.3
DETR	0.781	0.750	0.466	41.2 M	48.1
the proposed one	0.813	0.791	0.555	17.3 M	137.7

**Table 5:** Detection precisions of the proposed method for different TI categories.

TI Categories	OA	F1-Score
V-TI	0.941	0.913
S-TI	0.899	0.870
F-TI	0.854	0.827

**Table 6:** Detection precisions of the proposed method on Sentinel-2 images.

OA	F1	mAP50	mAP75
0.831	0.802	0.567	0.485

## Data Availability

No new data were created or analyzed in this study. Data sharing is not applicable to this article.
